# Family medicine journals’ endorsement of reporting guidelines and clinical trial registration: a cross-sectional analysis

**DOI:** 10.3399/BJGPO.2023.0183

**Published:** 2024-10-30

**Authors:** Wyatt Compton, Brody Dennis, Payton Clark, Caleb A Smith, Danya Nees, Griffin Hughes, Matt Vassar

**Affiliations:** 1 Office of Medical Student Research, Oklahoma State University Center for Health Sciences, Tulsa, OK, US; 2 Department of Psychiatry and Behavioral Sciences, Oklahoma State University Center for Health Sciences, Tulsa, OK, US

**Keywords:** reporting guidelines, clinical trial registration, research quality

## Abstract

**Background:**

Family medicine, vital for patient care but underfunded, prompts an evaluation of how family medicine journals endorse, require, and advocate for reporting guidelines (RGs), clinical trial, and systematic review registration.

**Aim:**

Assess endorsement and requirement of RGs, and the stance on registration of clinical trials and systematic reviews in family medicine journals, impacting research quality and transparency.

**Design & setting:**

A cross-sectional analysis of 43 ‘family practice’ journals, identified through the 2021 Scopus CiteScore, was undertaken. Editors-in-chief were contacted to confirm article types. Data extracted from ‘instructions to authors’ pages focused on recommendations or requirements for use of RGs, and for trial registration.

**Method:**

To ensure confidentiality and prevent bias, authors independently extracted data on the requirement or recommendation for use of RGs and clinical trial registration to provide an overview of research standards.

**Results:**

From the 43 journals, the most recommended guidelines were CONSORT (69%), PRISMA (58%), and STROBE (60%). The most required were PRISMA (16%) and CONSORT (11%). Clinical trial registration was recommended or required by 67% of journals. Additionally, 40 out of the 43 (93%) journals cited at least one reporting guideline in their instructions to authors.

**Conclusion:**

Family medicine journals exhibit a variety of endorsement and requirement patterns for RGs and clinical trial registration. While guidelines like CONSORT, PRISMA, and STROBE are acknowledged, caution is needed in presuming a direct link between mention of these RGs and enhanced research quality. A nuanced approach, promoting diverse RGs and rigorous study registration, is essential for elevating transparency and advancing research standards in family medicine.

## How this fits in

Before this research, the endorsement and requirement of reporting guidelines (RGs) and clinical trial registration in family medicine journals was not well documented. RGs and trial registration are valuable tools that can improve research quality and reduce bias. This study adds critical insights by revealing that while some family medicine journals endorse RGs and clinical trial registration, many do not, potentially impacting the quality and transparency of research in this field. Clinicians should be aware of this variability in practice to better evaluate the quality and reliability of findings within the field of family medicine.

## Introduction

Every year, >7 million scientific articles are published across >20 000 academic journals, with publication rates varying by field and specialty.^1^ Presently, the field of family medicine has 45 actively publishing journals with a Scopus CiteScore, a relatively small number compared to domains like internal medicine, which boasts 143 active journals with CiteScores on the same database.^2^ Family medicine is a broad and important area of medicine, with >100 000 physicians in the US practicing in the field.^3^ Family medicine physicians provide the highest number of healthcare encounters in the US, such as managing terminally ill patients, identifying health threats within a community, and advocating for accessible health care for all.^4,5^ Given the extensive scope of family medicine and its contribution to patient care, robust medical research in this field is essential. Between 2002 and 2006, family medicine departments received $187 million in grants from the National Institutes of Health, accounting for only 0.20% of the total $95.3 billion awarded during that period.^5^ This disparity underscores the need for a focused examination of research practices in family medicine, particularly in the context of RGs and clinical trial registration.

RGs serve as checklists for researchers and authors when conducting a specific type of research study design. These guidelines facilitate reporting of the information needed in an article to be used as strong evidence for clinical decisions, to be reproduced by other researchers, and to be understood by readers.^4^ One example of an RG is the Consolidated Standards of Reporting Trials (CONSORT) used to mitigate bias and increase transparency in clinical trials. Plint *et al* found that when journals endorsed the CONSORT RG, the amount of reported data items and the quality of research increased.^6^ Likewise, Panic *et al* determined that when journals endorsed the Preferred Reporting Items for Systematic Reviews and Meta-Analyses (PRISMA) guideline, both methodological and reporting quality of published studies improved.^7^ To further champion the dissemination of RGs, the Enhancing the QUAlity and Transparency Of health research (EQUATOR) Network was established with the goal of maximising the transparency and reporting quality of clinical research (https://www.equator-network.org). To date, the EQUATOR Network hosts over 500 unique RGs for various study designs including CONSORT, PRISMA, and Strengthening the Reporting of Observational Studies in Epidemiology (STROBE). However, it is essential to understand that while RGs contribute to comprehensive reporting, they are not direct indicators of research quality or a measure for reducing bias.^8^

In addition to the use of RGs, another strategy to improve transparency of study results is the requirement for clinical trial registration. Prospective clinical trial registration has been associated with a reduced risk of bias. A study of paediatric randomised controlled trials found that those with incomplete adherence to trial registration had a higher risk of bias sequence generation and allocation concealment when compared to prospectively registered trials.^9^ Additionally, Lindsley *et al* found an association with increased reporting bias, detection bias, performance bias, allocation concealment, and random sequence generation with unregistered clinical trials.^10^ Despite this association, there is evidence that bias continues to be a central issue in medical research. An extensive review by McGauran *et al* identified multiple types of reporting bias in clinical trials across 50 different interventions (pharmacological, diagnostic, surgical, and preventative interventions).^11^ Jones *et al* found many trials have discrepancies in the primary outcomes between registration and publication, with some as great as 50%.^12^ Prospective registration of clinical trials is a fundamental step in ensuring transparency and reproducibility of studies, yet it is often overlooked. In an effort to address these issues, the International Committee of Medical Journal Editors (ICMJE), an organisation dedicated to improving medical research practices, mandates that any clinical trial to be published in their network of journals must be registered before starting the study.^13^

Academic journals can play a central role in the advocacy of RGs and prospective clinical trial registration by requiring or recommending the use of these tools within their ‘instructions to authors’ webpages. However, to our knowledge, the rate of recommendation or requirement of RGs and clinical trial registration by family medicine journals has not been thoroughly studied in the current literature. Family medicine, encompassing a wide range of healthcare services, significantly influences patient outcomes. Research quality and transparency in this field are not merely academic concerns but have direct implications for patient care.^14^ Thus, the goal for the present study is to evaluate the rate at which family medicine journals advocate for the use of RGs and clinical trial registration.

## Method

### Study design

Using the STROBE checklist, we conducted a cross-sectional analysis of family medicine journals’ policies and guidelines for authors.^15^ Our protocol, raw data, analysis scripts, extraction forms, and standardised email prompts were uploaded to Open Science Framework (OSF) in order to promote transparency and reproducibility.^16^

### Search strategy

With the aid of a medical research librarian, all clinical journals within the subcategory of ‘family practice’ were identified using the 2021 Scopus CiteScore tool as our exclusive database for journal selection.^2^ Scopus was chosen for its comprehensive coverage and relevance to family medicine, ensuring consistency and uniformity in our dataset. This decision aligns with the specific scope of our research, focusing on a well-defined subcategory where Scopus provides the most relevant and comprehensive information. To validate the accuracy of the top 20 journals identified by Scopus, we cross-referenced them using the h5-index provided by Google Scholar Metrics, which is a tool with extensive coverage of scholarly journals and the ability to capture a broader range of citation metrics.^17^ This provides an additional layer of verification to enhance the robustness of our journal selection process. During the review process on Scopus, we followed standard practices for literature review and journal identification. It is important to note that Scopus itself does not provide specific guidelines for conducting reviews within its interface. In line with our goal of inclusivity, we did not apply any additional limiters during the search process. This approach was adopted to provide a comprehensive representation of currently active clinical journals in family medicine, and it ensures transparency in our methodology.

### Eligibility

All currently active journals that publish clinical research regarding family medicine were eligible for inclusion in our study. If a journal was written in a language other than English, we used Google Translate to translate the language into English.^18^

### Exclusion criteria

Discontinued journals were excluded to prevent the unfair assessment of inactive journals. Journals that lacked clear contact information for communication with the editorial office were also excluded. This decision was made to avoid evaluating journals without the opportunity to seek input on the journal’s publication policies from the editorial team. Finally, academic books were excluded from our sample.

### Data collection process

For each journal, two investigators (WC, BD) extracted data from the ‘instructions to authors’ webpage in a masked, duplicate fashion and recorded this data in a standardised Google Form designed by investigators CAS, DN, and MV. On completion of extraction, the two investigators (WC, BD) were unmasked to reconcile one another’s data and resolve any discrepancies. If a discrepancy was unable to be resolved, a third investigator (PC) was consulted.

### Data items

Extracted data items from each journal included the journal editor response rate, journal title, 5-year impact factor, whether EQUATOR Network was mentioned, ICMJE acknowledgements in the instructions to authors, and continent of journal publication. ICMJE acknowledgments refers to a statement by the journal in which they endorse the criteria for ethical and transparent reporting of research set out by the ICMJE. We extracted statements for each journal’s instructions to authors in regard to RGs. A description of the RGs of interest can be found in Table 1.

**Table 1.** Reporting guidelines and study designs

Study design	Respective reporting guideline
Animal research	ARRIVE
Case reports	CARE
Clinical trials	CONSORT
Clinical trial protocols	SPIRIT
Diagnostic accuracy	STARD
	TRIPOD
Economic evaluations	CHEERS
Observational studies in epidemiology	MOOSE
	STROBE
Qualitative research	COREQ
	SRQR
Quality improvement	SQUIRE
Systematic reviews and meta-analyses	PRISMA
	QUOROM
Systematic review and meta-analysis protocols	PRISMA-P

ARRIVE = Animal Research: Reporting of In Vivo Experiments. CARE = CAse REports guidelines checklist. CHEERS = Consolidated Health Economic Evaluation Reporting Standards. CONSORT = Consolidated Standards of Reporting Trials. COREQ = Consolidated Criteria for Reporting Qualitative Research. MOOSE = Meta-Analysis of Observational Studies in Epidemiology. PRISMA = Preferred Reporting Items for Systematic Reviews and Meta-Analyses. PRISMA-P = Preferred Reporting Items for Systematic Review and Meta-Analysis Protocols. QUOROM - Quality of Reporting of Meta-analyses. SPIRIT = Standard Protocol Items: Recommendations for Interventional Trials. SQUIRE = Standards for QUality Improvement Reporting Excellence. SRQR = Standards for Reporting Qualitative Research. STARD = Standards for Reporting of Diagnostic Accuracy Studies. STROBE = Strengthening the Reporting of Observational Studies in Epidemiology. TRIPOD = Transparent Reporting of a multivariable prediction model for Individual Prognosis Or Diagnosis.

Additionally, we extracted statements from each journal about clinical trial registration in databases including ClinicalTrials.gov, World Health Organization International Clinical Trial Registry Platform, the International Prospective Register of Systematic Reviews (PROSPERO), or any other trial registry.

We categorised each given guideline or study registry policy as required or compulsory, recommended, or not required or not mentioned. Guidelines and study registries with phrases such as ‘required’, ‘must’, ‘need’, ‘mandatory’, and ‘studies will not be considered for publication unless … ’ were interpreted as ‘required’. Phrases such as ‘recommended’, ‘encouraged’, ‘should’, and ‘preferred’ were interpreted as ‘recommended’. If language was unclear, categorisation was decided in consensus between study investigators. If a journal directly referred readers to the EQUATOR Network for guideline adherence instead of listing guidelines individually, we assumed that relevant guidelines for each included article type were considered.

To prevent the unfair assessment of journals for article types they may not accept, the editorial team of each journal was contacted via email about accepted article types. For each journal, the correspondent was emailed once a week for 3 weeks using a standardised email prompt designed by CAS. If no response was received after 3 weeks, no judgements were drawn in regard to the accepted article types and the journal was scrutinised against all data items assessed.

### Investigator training

Before beginning data extraction, authors (WC, BD) underwent training to ensure consistent interpretation of scope, rationale, and implementation of methods led by CAS, DN, and MV. After training, authors (WC, BD) underwent a practice exercise that involved data extraction from five journals not in our sample set in a masked, duplicate manner. When the data extraction was complete, the authors (WC, BD) were unmasked and discussed any discrepancies. Once this training was finished, authors (WC, BD) began data extraction from the full study sample.

### Outcomes

Our primary outcome for this study was to measure the proportion of family medicine journals’ instructions to authors that recommend or require the use of reporting guidelines relevant to common biomedical study designs. Our secondary outcome was to record the proportion of journals that recommend or require clinical trial registration.

### Data synthesis

Data summarisation was carried out via R (version 4.2.1) and RStudio (version 2023.03.2+454). The following information was synthesised: frequencies and percentages of guidelines referenced in the study sample and the frequencies and percentages of journals referencing clinical trial registration. Because this research is an assessment of simple frequencies at the level of academic journals rather than individual studies, an analysis for bias was not required during the production of this article.

## Results

### Journal characteristics

This cross-sectional analysis identified 45 family medicine journals from Scopus CiteScore. The journal *Asia Pacific Family Medicine* was excluded from the study because its publication had been discontinued. In addition, *Family Practice Management* was excluded after an email response from an editor indicated that the journal did not focus on research in family medicine. In total, 43 journals were included for data extraction. The final dataset of included journals can be found in Supplementary Table S1.

The 5-year impact factor for included journals ranged from 0.42 to 6.92 (mean 3.20, standard deviation [SD] 1.83). There were 27 journals with no listed 5-year impact factor at the time of analysis. In terms of geographical distribution of editorial offices, 19 were in Europe (44%), 12 were in Asia (28%), six were in North America (14%), and six were classified as ‘other’ (14%). Email inquiries were given 3 weeks for responses; after 3 weeks, only nine of 43 (21%) journals had responded. Study designs not accepted by individual journals are described in Supplementary Table S1 and were excluded from the analysis.

### Reporting guidelines

In our analysed sample, three journals (7%) did not mention RGs. Twenty-three journals (53%) made reference to the EQUATOR Network, and 30 journals (70%) included an ICMJE statement. The most frequently cited reporting (required and recommended) guidelines were CONSORT (81% of journals), followed by PRISMA (74%) and STROBE (62%). The least referenced reporting guideline was QUOROM (5%). The least referenced reporting guideline was QUOROM (5%). Guidelines like CHEERS, PRISMA-P, and TRIPOD were each referenced by three journals (7%), while MOOSE received four mentions (9%). For a comprehensive breakdown of individual journal policies concerning reporting guidelines, please refer to Supplementary Table S1.

### Clinical trial registration

In terms of clinical trial registration, 14 journals failed to mention clinical trial registration policy (33%). Twenty-four journals required registration (56%; Table 2), four journals recommended registration (9%), and one journal did not accept clinical trials (2%).

**Table 2.** Journals requiring prospective registration of trials

*African Journal of Primary Health Care and Family Medicine*
*Anatolian Journal of Family Medicine*
*Annals of Family Medicine*
*Atención Primaria*
*Atención Primaria Práctica*
*Australian Journal of General Practice*
*BMC Primary Care*
*Canadian Family Physician*
*Computer Assisted Surgery*
*Eurasian Journal of Family Medicine*
*European Journal of General Practice*
*Family Medicine*
*Family Medicine and Community Health*
*International Journal of Community Based Nursing and Midwifery*
*Journal of Family and Community Medicine*
*Journal of Family Nursing*
*Malaysian Family Physician*
*Osteopathic Family Physician*
*Pediatria i Medycyna Rodzinna*
*Practitioner*
*Revista Cuidarte*
*Semergen*
*Terapevticheskii Arkhiv*
*The Lancet Healthy Longevity*

## Discussion

### Summary

Our examination revealed that the majority of family medicine journals did not explicitly refer to the use of RGs, except for CONSORT, PRISMA, and STROBE. Slightly more than half of the journals required clinical trial registration, while one-third of them did not mention it. It is unsurprising that QUOROM had the lowest likelihood of being required or recommended, given its replacement by PRISMA in 2005, as documented in prior literature.^19,20^ Notably, the EQUATOR Network was cited by 51% of the journals included in our study. Additionally, one-third of the journals lacked an ICMJE statement. These findings collectively suggest that, while a substantial number of journals incorporated ICMJE statements and referenced the EQUATOR network, a significant proportion failed to acknowledge or advocate for the majority of existing RGs. This could impact the comprehensiveness of reporting, although not necessarily the quality or bias of research outcomes.

### Strengths and limitations

This study has many strengths. First, this study was conducted based on a protocol designed a priori. Second, the data extraction process was conducted in a masked, duplicate manner, which is the ‘gold standard’ technique for studies involving the extraction of data from publications. Finally, all materials relevant to the conduction of this study have been uploaded on the public platform OSF for the purposes of transparency and reproducibility.^16^ This study is not without limitations, however. Despite our efforts to communicate with journal editorial teams, we had poor levels of response. This may have limited the accuracy of our interpretations of journal webpages and policies for publication. Furthermore, the cross-sectional nature of our study limits the generalisability of our findings to this specific point in time as journal policies, editorial practices, and federal laws are often subject to change.

### Comparison with existing literature

Previous studies highlight the benefits of adherence to RGs. A study performed by Moher *et al* analysed the benefits of adherence to CONSORT guidelines across four journals over 4 years (1994–1998).^21^ In this study, three of the journals that adopted CONSORT guidelines (*BMJ*, *JAMA*, and *The Lancet*) were compared to one journal that did not (*The New England Journal of Medicine*). It was found that the three journals that endorsed the CONSORT guidelines had a statistically significant improvement in the quality score reports for randomised controlled trials over the journal that did not adopt CONSORT guidelines. This study demonstrates that adherence to CONSORT guidelines may improve the quality of research reporting. Of the 43 journals we sampled, only six required that authors adhere to CONSORT (see Supplementary Table S1). Meerpohl *et al* found that only one-fifth of paediatric journals mentioned CONSORT, with only three requiring adherence.^22^ Thus, while family medicine journals mention CONSORT at a higher rate than paediatric journals, there is still room for improvement.

Similar findings regarding journal endorsement have been observed with PRISMA for systematic reviews. Nawijn *et al* ^23^ assessed the reporting quality of systematic reviews and meta-analyses in emergency medicine using the PRISMA statement. The top five emergency medicine journals were scrutinised for systematic reviews and meta-analyses published between 2015 and 2016. While reviews indicating PRISMA adherence did not consistently show better reporting than non-adherent reviews, journals that mandated reporting guideline adherence exhibited higher reporting quality. This finding suggests that active enforcement of reporting guidelines, such as requiring PRISMA endorsement, can enhance the overall reporting standards in research.

Clinical trial registration is required by federal law in the US but many studies still fail to prospectively register their trials, resulting in bias.^24^ A study comparing the registered and published primary outcomes in randomised controlled trials found that less than half of the trials adequately registered their trial with clear primary outcomes.^25^ Lack of proper registration can lead to selective outcome reporting, which is where changes to the outcomes reported favour statistically significant results rather than statistically non-significant results.^25^ Similarly, a study by Killeen *et al* analysing surgical randomised controlled trials found that discrepancies in published primary outcomes and registered primary outcomes arose when studies failed to prospectively register their trials.^26^ Requiring clinical trial registration is essential to prevent publication bias and selective outcome reporting bias, as well as maximise transparency of clinical trials.^10^ Our study shows that approximately half of the journals surveyed require clinical trials to be registered. All family medicine journals should require prospective registering of clinical trials, as unregistered trials have been shown more likely to display favourable or biased results.^10^ Among other barriers, clinical trial registration may be a reason that some authors submit their studies to journals with less strict requirements. However, clinical trial registration should be strictly enforced to ensure accountability and study reproducibility within the field of family medicine.

### Implications for research and practice

This study’s findings have notable implications for future research and clinical practice in the field of family medicine. The observed limited incorporation of RGs, with explicit mentions primarily centred around CONSORT, PRISMA, and STROBE, suggests a potential area for improvement in the reporting standards of family medicine journals. While it is important to note that adherence to RGs does not necessarily equate to assured research quality, promoting their wider adoption could positively influence the comprehensiveness of reporting. This cautious interpretation aligns with the understanding that RGs serve as guides for comprehensive reporting rather than direct indicators of research quality or bias reduction. Additionally, the identified lack of emphasis on clinical trial registration in a substantial portion of the surveyed journals indicates a gap in ensuring transparency and minimising bias in clinical trial reporting. Encouraging family medicine journals to consider and, where appropriate, enforce prospective clinical trial registration remains crucial for preventing publication bias and enhancing study reproducibility. The study’s recommendations for promoting awareness of RGs and clinical trial registration emphasise a nuanced perspective, acknowledging their role in comprehensive reporting without presuming a direct link to research quality.

To conclude, this cross-sectional analysis of the instructions to authors of family medicine journals found that many journals lack guidance for prospective authors regarding adherence to RGs. Approximately one-third of these journals do not mention clinical trial registration. To enhance research reporting and reduce bias in family medicine, journals should be encouraged to enforce appropriate RGs and prospective clinical trial registration, although it is important to note that adherence to RGs enhances reporting transparency and should not be equated with research quality or bias reduction.

## Data

The protocol, raw data, analysis scripts, extraction forms, and standardised email prompts for this study are publicly available on the Open Science Framework to promote transparency and reproducibility. The dataset can be accessed at the following repository:

Repository name: Family Medicine Journals’ Endorsement of Reporting Guidelines and Clinical Trial Registration

DOI: 10.17605/OSF.IO/DRQ69

URL: https://osf.io/drq69/?view_only=3dbdb13cdeb545a8a0256ecd6f7e49f9
